# Nitrogen application enhances yield, yield-attributes, and physiological characteristics of dryland wheat/maize under strip intercropping

**DOI:** 10.3389/fpls.2023.1150225

**Published:** 2023-03-22

**Authors:** Sadam Hussain, Muhammad Asad Naseer, Ru Guo, Fei Han, Basharat Ali, Xiaoli Chen, Xiaolong Ren, Saud Alamri

**Affiliations:** ^1^ College of Agronomy, Northwest A&F University, Yangling, Shaanxi, China; ^2^ Institute of Water Saving Agriculture in Arid Areas of China, Northwest A&F University, Yangling, Shaanxi, China; ^3^ Key Laboratory of Crop Physic-ecology and Tillage Science in Northwestern Loess Plateau, Ministry of Agriculture, Northwest A&F University, Yangling, Shaanxi, China; ^4^ Institute of Crop Science, University of Bonn, Bonn, Germany; ^5^ Department of Agricultural Engineering, Khwaja Fareed University of Engineering and Information Technology, Rahim Yar Khan, Pakistan; ^6^ Department of Botany and Microbiology, College of Science, King Saud University, Riyadh, Saudi Arabia

**Keywords:** co-growth period, crop rows, nitrogen, physiology, productivity, SPAD

## Abstract

Intercropping has been acknowledged as a sustainable practice for enhancing crop productivity and water use efficiency under rainfed conditions. However, the contribution of different planting rows towards crop physiology and yield is elusive. In addition, the influence of nitrogen (N) fertilization on the physiology, yield, and soil water storage of rainfed intercropping systems is poorly understood; therefore, the objective of this experiment was to study the contribution of different crop rows on the physiological, yield, and related traits of wheat/maize relay-strip intercropping (RSI) with and without N application. The treatments comprised of two factors viz. intercropping with three levels (sole wheat, sole maize, and RSI) and two N application rates, with and without N application. Results showed that RSI significantly improved the land use efficiency and grain yield of both crops under rainfed conditions. Intercropping with N application (+N treatment) resulted in the highest wheat grain yield with 70.37 and 52.78% increase as compared with monoculture and without N application in 2019 and 2020, respectively, where border rows contributed the maximum followed by second rows. The increase in grain yield was attributed to higher values of the number of ears per square meter (10-25.33% more in comparison to sole crop without N application) during both study years. The sole wheat crop without any N application recorded the least values for all yield-related parameters. Despite the absence of significant differences, the relative decrease in intercropped maize under both N treatments was over 9% compared to the sole maize crop, which was mainly ascribed to the border rows (24.65% decrease compared to the sole crop) that recorded 12 and 13% decrease in kernel number and thousand-grain weight, respectively than the sole crop. This might be attributed to the reduced photosynthesis and chlorophyll pigmentation in RSI maize crop during the blended growth period. In a nutshell, it can be concluded that wheat/maize RSI significantly improved the land use efficiency and the total yield compared to the sole crops’ yield in arid areas in which yield advantages were mainly ascribed to the improvement in wheat yield.

## 1 Introduction

The world’s burgeoning population (FAO et al., 2019), consequences of climate change (Challinor et al., 2014), and water shortage (Varis et al., 2017) are acknowledged to be the major challenges for global food security. Cereal grains are consumed as staple foods in many parts of the world. Therefore, their production is to be increased to meet the food needs of the increasing population (Grote et al., 2021). Furthermore, improved food security will also depend on the judicious use of available resources including land, water, and nutrients (Vågsholm et al., 2020). Rainfed agriculture is one of the major water-saving practices which occupy more than 80% of farmland area in the world and 60% in East- and South- Asia (Wani et al., 2009; David et al., 2011). According to an estimate, about 60% of world grains are produced from rainfed areas (UNESCO, 2009). However, the yield of crops in rainfed areas is affected severely under changing climatic scenarios (Bakhsh and Kamran, 2019). Several approaches, including mixed cropping practices like intercropping, have been reported as widespread land management practice to enhance resource use efficiency. These can be used for achieving higher yield and productivity under rainfed conditions (Li et al., 2020; Waha et al., 2020).

Intercropping, growing two or more crops simultaneously in the same field, has been reported as a sustainable agronomic approach that not only promotes crop growth but also boosts grain yield (Maitra et al., 2021). Many advantages have been demonstrated with intercropping including better soil quality (Roohi et al., 2022; Wolińska et al., 2022), enhanced microbial populations (Obi et al., 2022; Zhao et al., 2022), reduced pest populations (Yang et al., 2022), high nutrient acquisition efficiency (Zhu et al., 2022), improved agronomic and physiological parameters (Jo et al., 2022) and overall greater crop yield (Brahimi et al., 2022). During the last few years, this practice has gained considerable attention in irrigated agriculture (Cuartero et al., 2022); various studies have reported a greater increase in grain yield under irrigated conditions (Cuartero et al., 2022; de Sá Souza et al., 2022). However, published reports demonstrated that this practice requires a huge amount of irrigation water (Jannoura et al., 2014). On the other hand, the unavailability of fresh water is one of the major yield-limiting factors in rainfed areas (Dotaniya et al., 2022). With the exception of recent studies (Erythrina et al., 2022; Jo et al., 2022), there are no published reports on intercropping under rainfed agriculture, particularly intercropping with dual cereal crops. In addition, previous studies mainly focused on strip intercropping (Alarcón-Segura et al., 2022; Jo et al., 2022), however, limited information exists on relay-strip-intercropping (Raza et al., 2022).

Wheat and maize are among the widely cultivated cereal crops, both in irrigated and rainfed agriculture. In the Loess Plateau of China, where rainfall is a major source of water for crop cultivation, strip intercropping of these crops is a common practice (Li et al., 2020). In intercropping, the “border effect” i.e., the ability of border rows to capture more inputs than others and yield more, is a common phenomenon (Li et al., 2020; Wang et al., 2021; Zhang et al., 2021). However, there is a lack of information on the contribution of inner and border rows towards crop yield, particularly, in wheat-maize intercropping systems. Intercropping significantly changes the canopy structure, which in turn can affect the ventilation, light interception, and leaf area (Li et al., 2021). Precious studies clearly demonstrated that inner rows intercept less sunlight when compared with border rows. There is a direct relationship between sunlight inception with crop photosynthesis rates. It is also well demonstrated that leaf area greatly influences the photoassimilates production and its supply to other organs which in turn can affect the yield of crops (Raza et al., 2022). Similarly, previously published reports have stated that changing the crop geometry alters the leaf area and, in this way, indirectly influences the production of photoassimilates for better growth and yield (Raza et al., 2022).

Nitrogen is an essential element for plant growth and development. Its application directly influences plant growth, development processes, plant nutrient cycling, and photosynthetic carbon (Zhang et al., 2007). Plants’ response to N application is highly dose-dependent (Liang et al., 2019). For example, in a recent study, Wang et al. (2022) demonstrated that increasing N rates decreased N fertilizer utilization by crop plants. During the last few years, most of the studies have discussed N’s influence on field-grown crops under a monocropping system. However, less attention has been devoted to the influence of N fertilization on the growth, physiological traits, and productivity of crops, particularly when grown in strip-intercropping. For this work, it was hypothesized that N application would improve crop physiology, yield, and land use efficiency in the wheat-maize intercropping system under rainfed conditions. The objectives of this study were to i) assess the effect of N fertilization on the physiology and yield performance of the rainfed intercropping system, ii) and evaluate the performance of border- and inner-rows in terms of physiological traits and yield of intercrops under N fertilization.

## 2 Materials and methods

### 2.1 Site description

**Figure 1:**
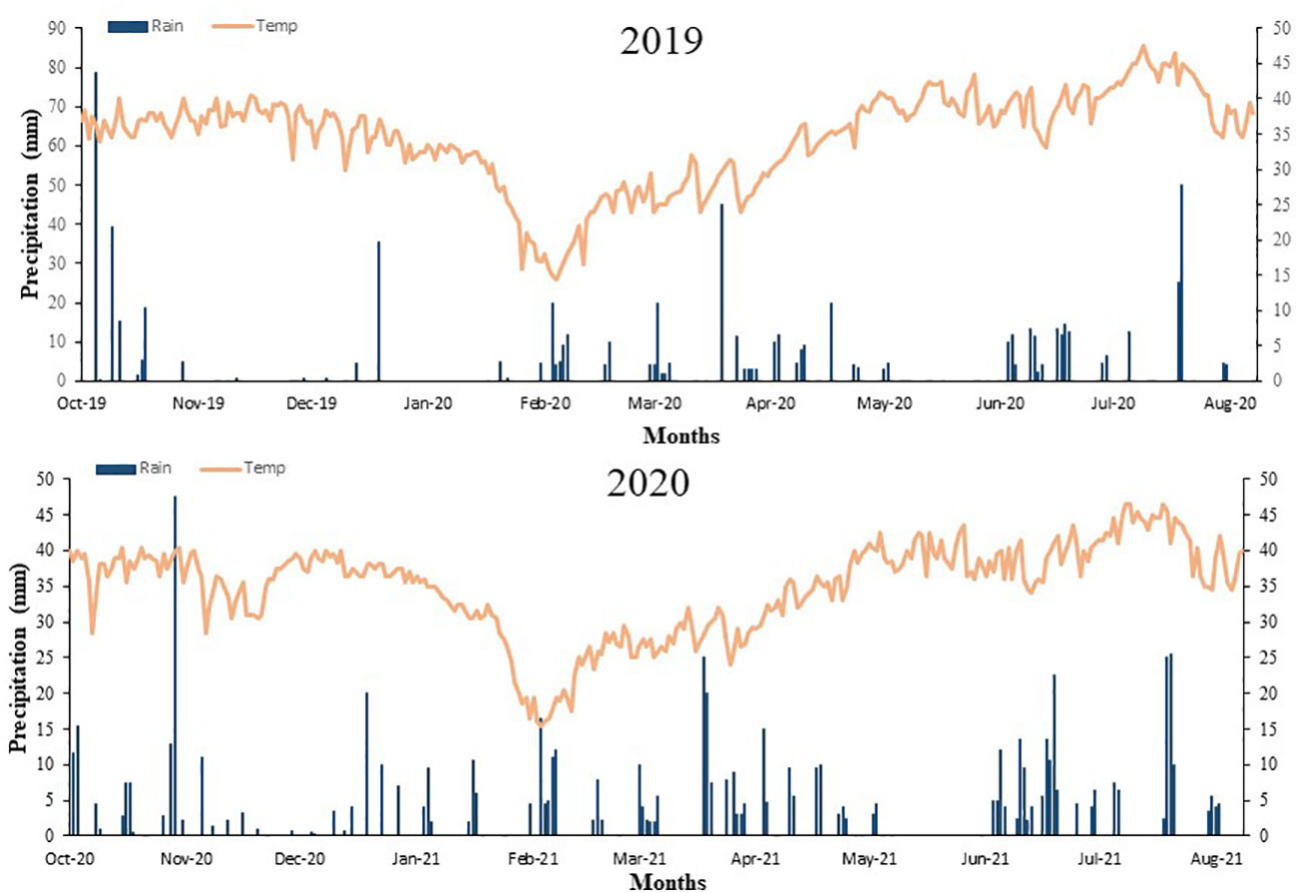
Daily weather data including the precipitation and average temperature during both experimental years.

The experimental site was established at Northwest Agriculture & Forestry University in 2019 and 2020 to explore the influence of wheat/maize intercropping and N treatments on photosynthetic and yield traits. The site at the experimental area is a loam soil with 26% field capacity and had been in under spring maize cultivation during the last three years. The meteorology data of the site location are from the nearest meteorological station and are given in **Figure 1**. The regional climate had the following properties: the yearly average temperature of 14.5°C and 575 mm of annual precipitation between 1970 and 2019, of which >65% of rainfall is concentrated from July to September. Pre-trial soil at the 0-30 cm layer had the following properties: 0.92 and 0.052, 0.015 and 0.096 g kg-1 of total N, available N, phosphorus (P), and potassium (K), respectively; 11.82 ± 0.5 of organic matter and 8.14 of soil pH.

### 2.2 Experimental design

**Figure 2:**
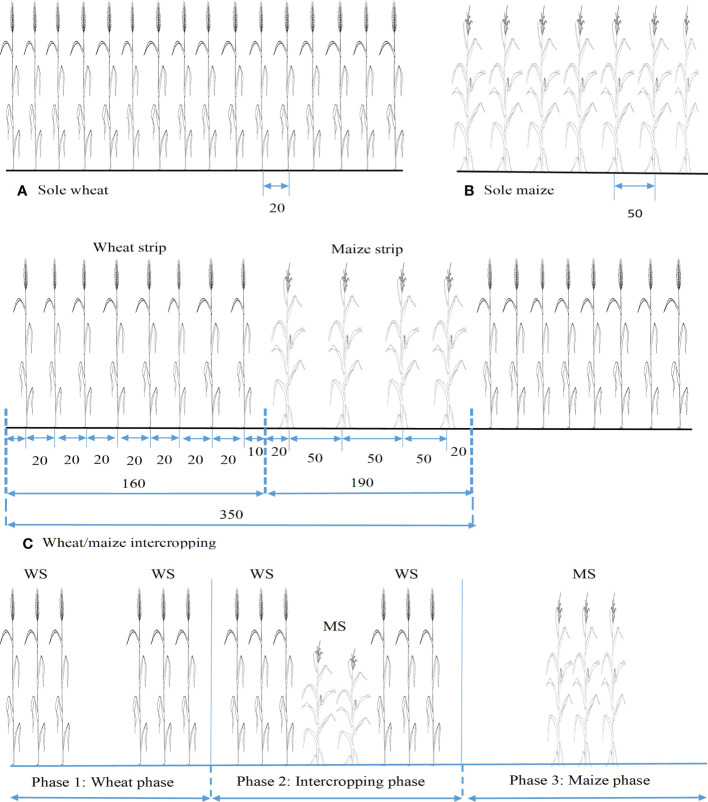
Layout of sole- **(A, B)** and strip- **(C)** intercropping of both crops.

The experimental field was divided following the randomized complete block design in which three intercropping viz. sole wheat, sole maize, and relay-strip-intercropping of both crops and two nitrogen levels viz. without- and with-N (basal application at 150 and 235 kg ha^−1^ for wheat and maize, respectively) application were maintained with three replicates. Each treatment plot was 10.5 m in length and 9 m in width comprising one meter follow area between the experimental plots which have north-to-south orientation for crop rows. Relay-strip-intercropping plots, with three strips in total, have eight and four rows of wheat and maize with 1.6 and 1.9 m widths, respectively in each strip. The whole planting geometry is given in **Figure 2**. The commonly cultivated wheat and maize cultivars viz. Yongliang 4 and Xianyu 335 were used with seedling rates of 180 kg ha^-1^ and 66,670 plants ha^-1^, respectively. A row spacing of 50, 30, and 20 cm was maintained for inter- and intra-maize and wheat crops, respectively for sole-crops and RSI treatments. Planting geometry was the same as the local practices (Ma et al., 2020). For RSI experimental unit, 30 cm distance was maintained between the adjacent crop rows. Wheat was sown on October 21 and October 13, and maize was sown on April 06 and March 30 during the first and second experimental years, respectively. The competitive growth phase between the two crops was about 2 months during both years. According to standard grower practice, phosphorus and potassium were applied at 176 and 40 kg ha^−1^ by using tricalcium phosphate {Ca_3_(PO_4_)_2_} and sulphate of potash, respectively. All fertilizers, including treated N, were applied as the basal dose of both crops under both sole- and RSI treatments. During both trial years, no irrigation was supplemented for sole crops and TSI treatments.

### 2.3 Data collection and analysis

#### 2.3.1 Yield and related traits

During both experimental years, at the maturity stage, plants from sole- and intercropped- rows were harvested from each experimental unit, containing eight and four rows (R) of wheat and maize, respectively, to obtain the yield and related traits. After manually threshing, the sun-dried grains of both crops were weighed. The ear number per meter (EN) in each row was counted. To calculate the kernels on each ear, approximately 20-40 ears/cobs per row were counted and then averaged. Furthermore, approximately 10-12 replications were harvested in each row to determine the thousand grains’ weight. In the current work, for RSI-wheat, R1 and R8 were described as border rows while adjusting rows viz. R2 and R7 were specified as 2^nd^ border rows. While R3 and R6, and R4 and R5 were characterized as 3^rd^ and center crop rows. Similarly, for the maize crop, under RSI system, R1 and R4 were described as the border while the remaining crop rows R2 and R3 were identified as the center crop rows. As opposed to RSI, we ignored the border effect for sole cropping treatments. For yield calculation, plants from the middle of the experimental area were harvested to reduce the experimental error.

#### 2.3.2 Photosynthetic attributes and SPAD values

A high efficient photosynthesis system LI-Cor, LI-6400XT was employed for Pn, Tr, and Gs measurement. For SPAD values, we used a dual-wavelength chlorophyll meter (SPAD 502, Minolta Camera Co., Ltd., Japan). For photosynthetic indices, an LED leaf chamber was used for the measurements. Furthermore, leaf water-use-efficiency was premeditated as the ratio of Pn and Tr. For that, we took the samples at about 10:00 a.m. on sunny days. For measurements, fully emerged top leaves were considered prior to the flag leaf stage and VT stage in wheat and maize, respectively. After that, flag- and ear leaves were used respectively in wheat and maize. For each experimental unit, the measurements were made by using approximately 10-12 leaves in the border- and center rows. The critical growth stages of both crops, as described in our previous study (Li et al., 2019), were considered for the measurements, including the SPAD value.

#### 2.3.3 Soil water storage

The soil moisture meter Diviner 2000 was used for assessing the soil water contents. For that, sampling was done from each 10 cm soil profile until the soil depth of 160 cm. The measurements were made during the critical crop growth phases, a detailed procedure is described in our previous study (Li et al., 2020). The soil water storage was determined as the multiplication of water content and soil depth, as previously reported by Li et al. (2020).

#### 2.3.4 Land equivalent ratio

We considered the partial land equivalent ratios of both crops (LRpw + LRpm), as previously described (Li et al., 2020), to estimate the total land equivalent ratio (Eq. 1). Measurements were made by considering the grain yield of both crops under the sole (Gyws and Gyms for wheat and maize, respectively) as well as strip-intercropping system (Gywi and Gymi for wheat and maize respectively), and planting ratios (Pw and Pm), using the equation as described by (Yu et al., 2015) with slight modifications.


1 $$\text{Land equivalent ratio}=\frac{Pw\;Gywi}{Gyws}+\frac{Pm\;Gymi}{Gyms}$$

### 2.4 Statistical analyses

Statistical Package for the Social Sciences (SPSS 20.0) software was used to perform the statistical analyses. One-way ANOVA was used for analyzing the mean difference at a 5% probability level. Correlation analyses were performed between grain yield and components for different rows, and between the Pn and yield components at various stages in both crops, and their means were differentiated by performing Duncan’s multiple range tests at *p*< 0.05.

## 3 Results

### 3.1 Yield and yield-related traits

Data showed that wheat-maize intercropping system non significantly affected the ear number per meter row, kernel number per ear, and thousand kernel weight of both crops during both study years under N treatments (with- and without N application), except for ear number per meter row for wheat in 2019 and 2020 (**Table 1**). Intercropping and N application resulted in 25.33 and 10.60% more number of wheat ears per meter row during 2019 and 2020, respectively in comparison to sole crop without N application. The sole wheat crop without any N application recorded the least values for all yield related parameters (**Table 1**).

**Table 1:** Effect of wheat-maize intercropping system on ear number per meter row (EN), kernel number per ear (KN), and Thousand kernel weight (TKW) during both study years under with- (+N) and with-out nitrogen (-N) application.

Year	Cropping system	N levels	EN (g)	KN (g)	TKW (g)
2019	Sole wheat	-N	400 c	30.96 a	32.4 a
		+N	425.16 bc	36.9 a	38.53 a
	Intercropped wheat	-N	485 ab	35.09 a	34.30 a
		+N	533.34 a	40.70 a	39.46 a
2020	Sole wheat	-N	411.67 d	34.66 a	31.03 b
		+N	455.33 c	40.16 a	36.33 ab
	Intercropped wheat	-N	505.66 b	32.8 a	33.94 ab
		+N	543.33 a	38.16 a	37.70 a
2019	Sole maize	-N	6.20 a	487 a	266.64 a
		+N	6.9 a	507.33 a	288.84 a
	Intercropped maize	-N	6.11 a	465 a	257.67 a
		+N	6.86 a	485 a	283.16 a
2020	Sole maize	-N	6.23 b	462 ab	246.34 a
		+N	6.70 a	487.67 a	268.5 a
	Intercropped maize	-N	6.24 ab	439.34 b	237.84 a
		+N	6.68 ab	457 ab	261.6 a

The means with the different lowercase letters are significantly different based on three-way anova analysis.

Wheat-maize intercropping system, N application and years significantly (P<0.001) affected the individual and total yield of both crops (**Table 2**). However, their interactive effect regarding yield was non-significant (**Table 2**). Results showed that intercropping with N application (+N treatment) resulted in the highest wheat grain yield with 70.37 and 52.78% increase as compared with monoculture and without N application in 2019 and 2020, respectively. However, sole plantation of maize and N application resulted in more yield. It was followed by intercropping and N application (**Table 2**). Likewise, intercropping with N application resulted in 33.60 and 29.80% more maize yield in 2019 and 2020, respectively in comparison to sole maize grown under N absence. Leaf equivalent ratios recorded for intercropping and N were significantly more during both years (**Table 2**).

**Table 2:** Effect of wheat-maize intercropping system on wheat and maize yields (t ha^-1^) and land equivalent ratios (LER) of averaged yield with- (+N) and with-out nitrogen (-N) application.

Year	Cropping system	N levels	Wheat	Maize	Total yield (g)	LER
2019	Monocropping	-N	4.86 d	8.11b	6.49	–
		+N	5.95 c	9.48a	7.71	–
	Intercropping	-N	7.17 b	7.82b	7.50	1.14
		+N	8.28 a	9.06a	8.67	1.17
2020	Monocropping	-N	5.21 d	6.86 c	6.04	–
		+N	6.02 c	8.29 a	7.16	–
	Intercropping	-N	7.20 b	6.34 d	6.77	1.12
		+N	7.96 a	7.71 b	7.84	1.15
Year			NS	***		
Cropping system			***	***		
Nitrogen			***	***		
Cropping system * Nitrogen			NS	NS		
Year * Cropping system * Nitrogen			NS	NS		

Weighted means of both crops in both systems i.e., intercropping, and sole cropping system, were expressed as the total yield. The yield of wheat and maize crops under intercropping treatment was the equivalence values of covered land area of each crop. The means with the different lowercase letters are significantly different based on three-way anova analysis. * Indicates p<0.05; ***, p<0.001; NS indicates non-significant.

**Figure 3:**
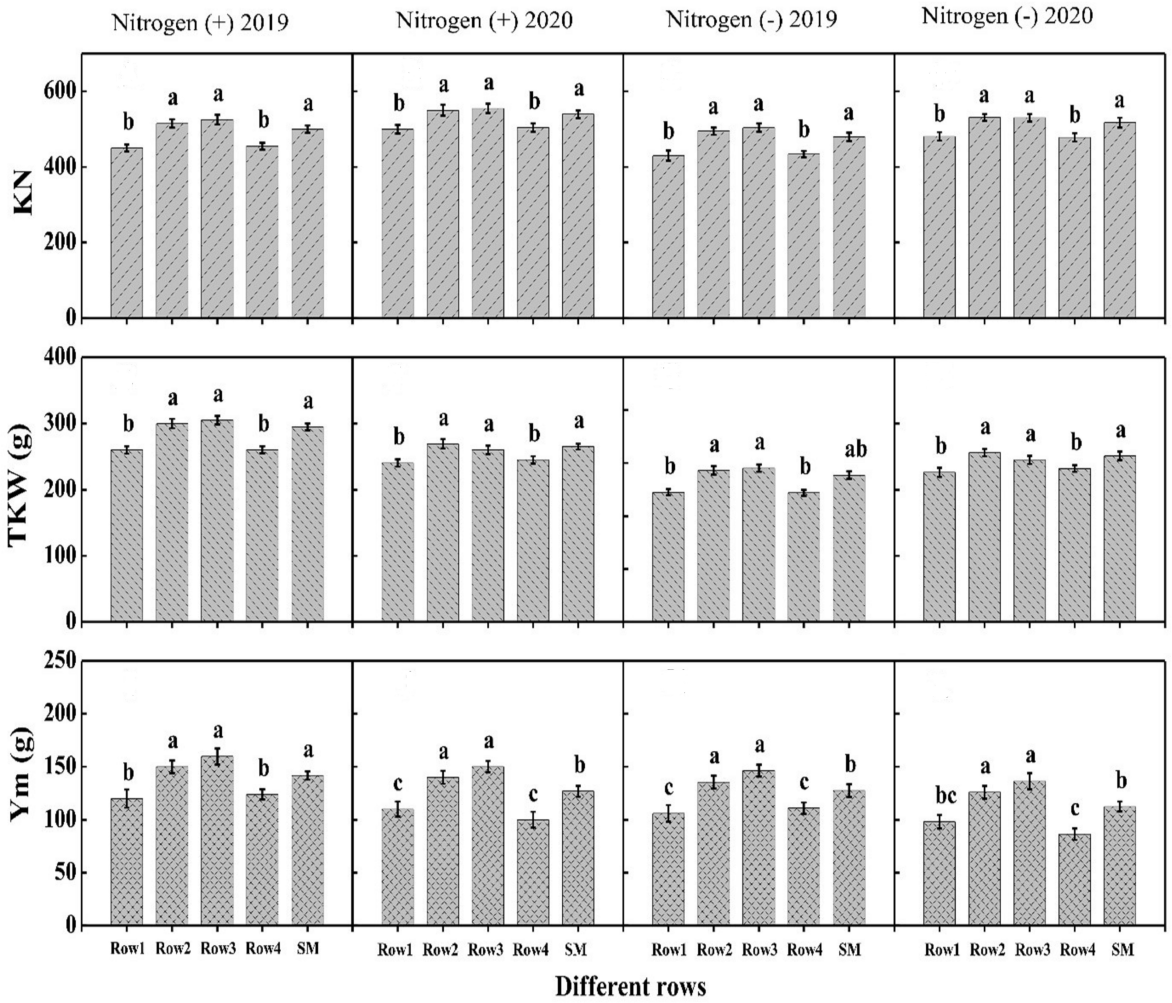
The kernel number per ear (KN), thousand kernel weight (TKW) and per meter grain yield (Ym, g) of different maize rows under with- and without N application. Different letters on the top of each bar indicate significant differences among the crop rows, as calculated by Tukey’s HSD test at P≤ 0.05.

**Figure 4:**
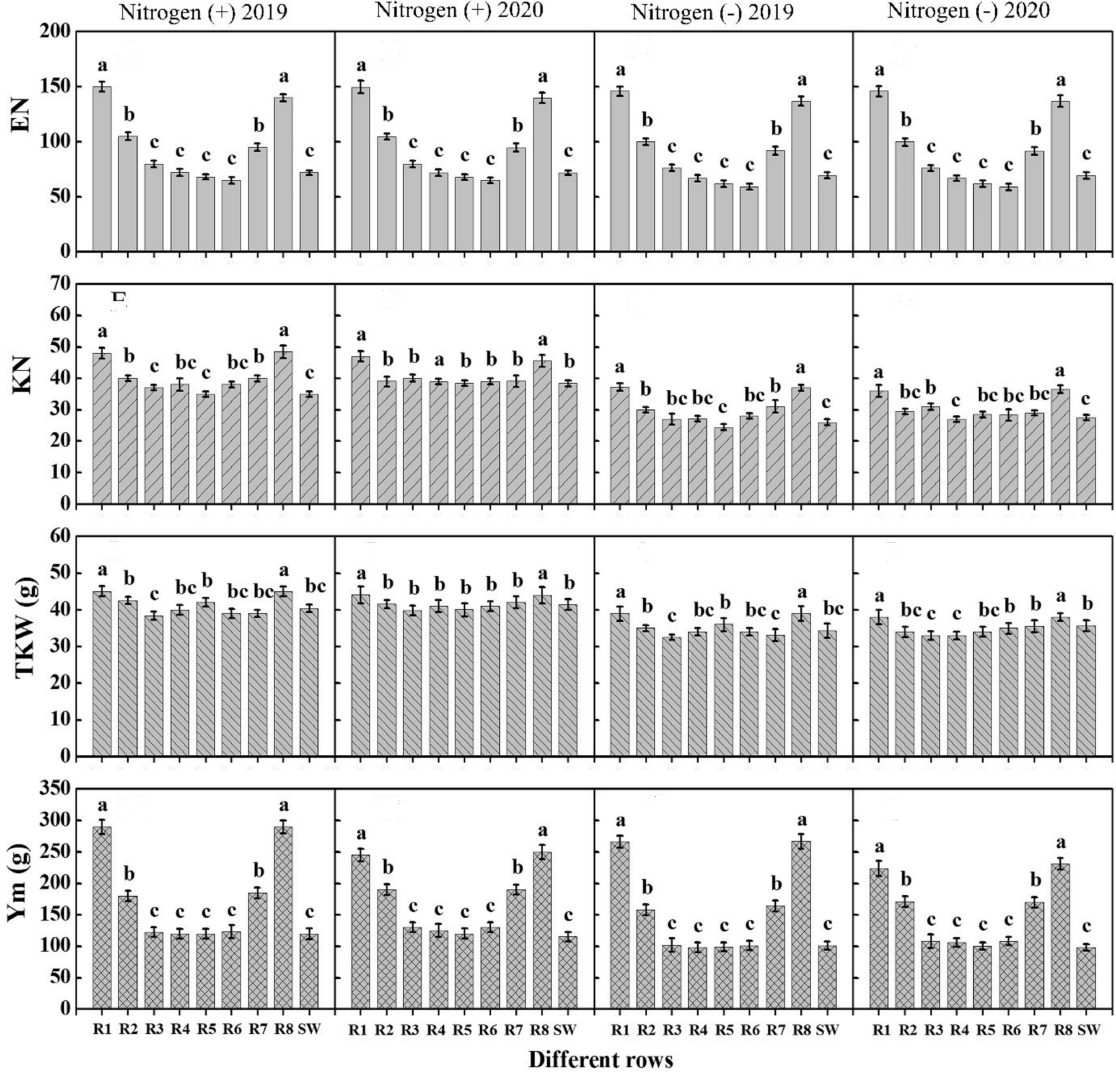
The kernel number per ear (KN), ear number per meter row (EN), thousand kernel weight (TKW, g) and per meter grain yield of different wheat rows under with- and without N application. Different letters on the top of each bar indicate significant differences among the crop rows, as calculated by Tukey’s HSD test at P≤ 0.05. R, row; SW, sole wheat.

Data regarding yield and related traits in maize showed significant difference among crop rows during both years and N treatments. Higher kernel number, thousand seed weight, and grain yield were recorded for Row-2 followed by Row-3 and the values were statistically at par (P<0.05) with sole maize rows during both years and N addition (**Figure 3**). The minimum values were depicted for Row-1 followed by Row-4 under both years and N treatments. For wheat, various rows performed differently with regard to ear number, kernel weight, thousand seed weight, and grain yield during both years and N treatments. Among crop rows, Row-1 depicted higher values of these traits, however, showed a statically similar response to Row-8 during both years and under both N rates (**Figure 4**). Further, Row-2 (R2) and Row-7 (R7) recorded lower values than border rows, however, significantly higher than those of Row-3 (R3), Row-4 (R4), Row-5 (R5), Row-6 (R6) as well as sole wheat rows which showed non-significant difference among each other and recorded lower values than other rows (**Figure 4**).

### 3.2 Leaf area index

**Figure 5:**
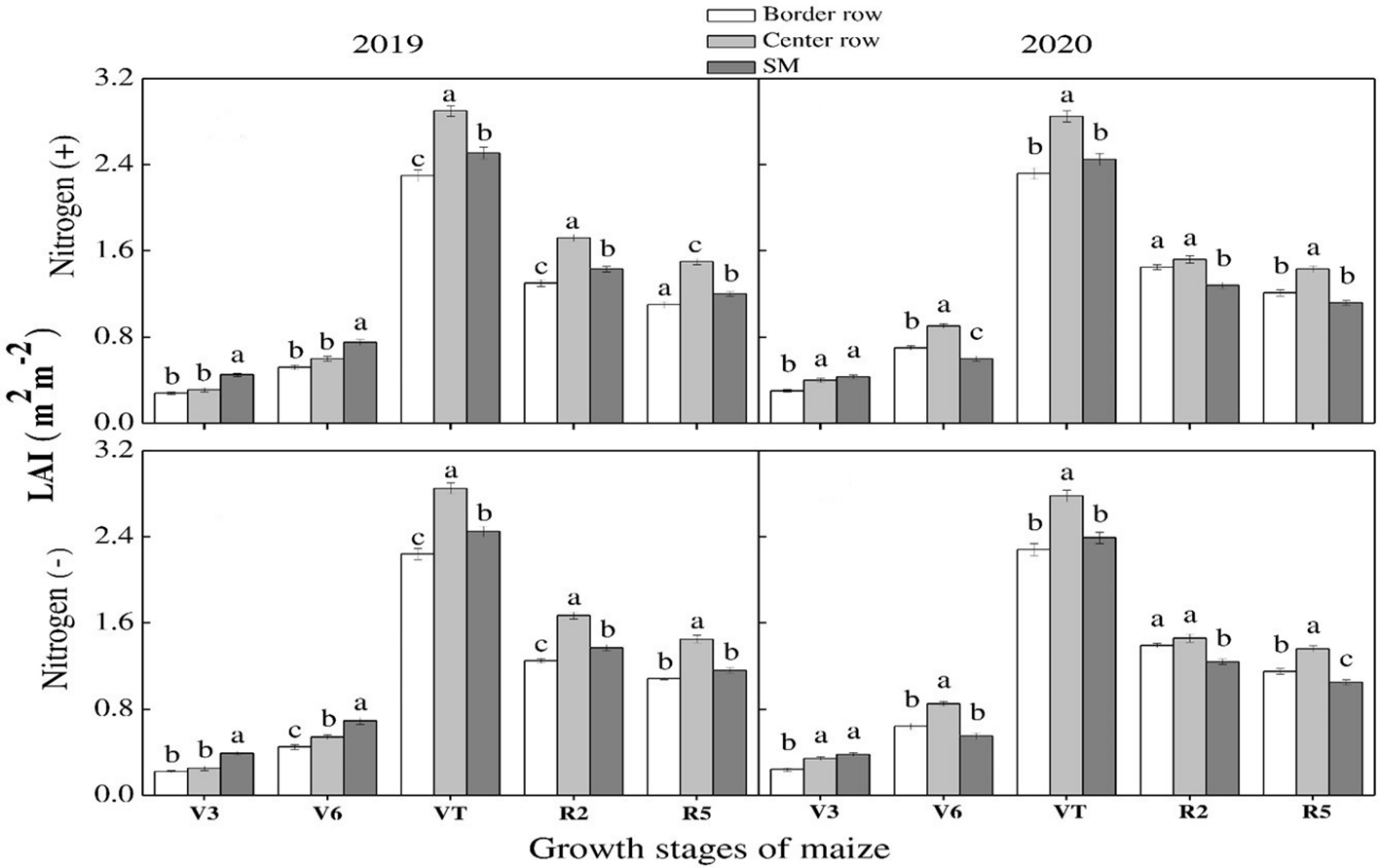
Leaf area index (LAI) of the border-, center- and sole maize (SM) crop at various growth stages under with- and without N application. Different letters on the top of each bar indicate significant differences among the various crop growth stages, as calculated by Tukey’s HSD test at P≤ 0.05.

**Figure 6:**
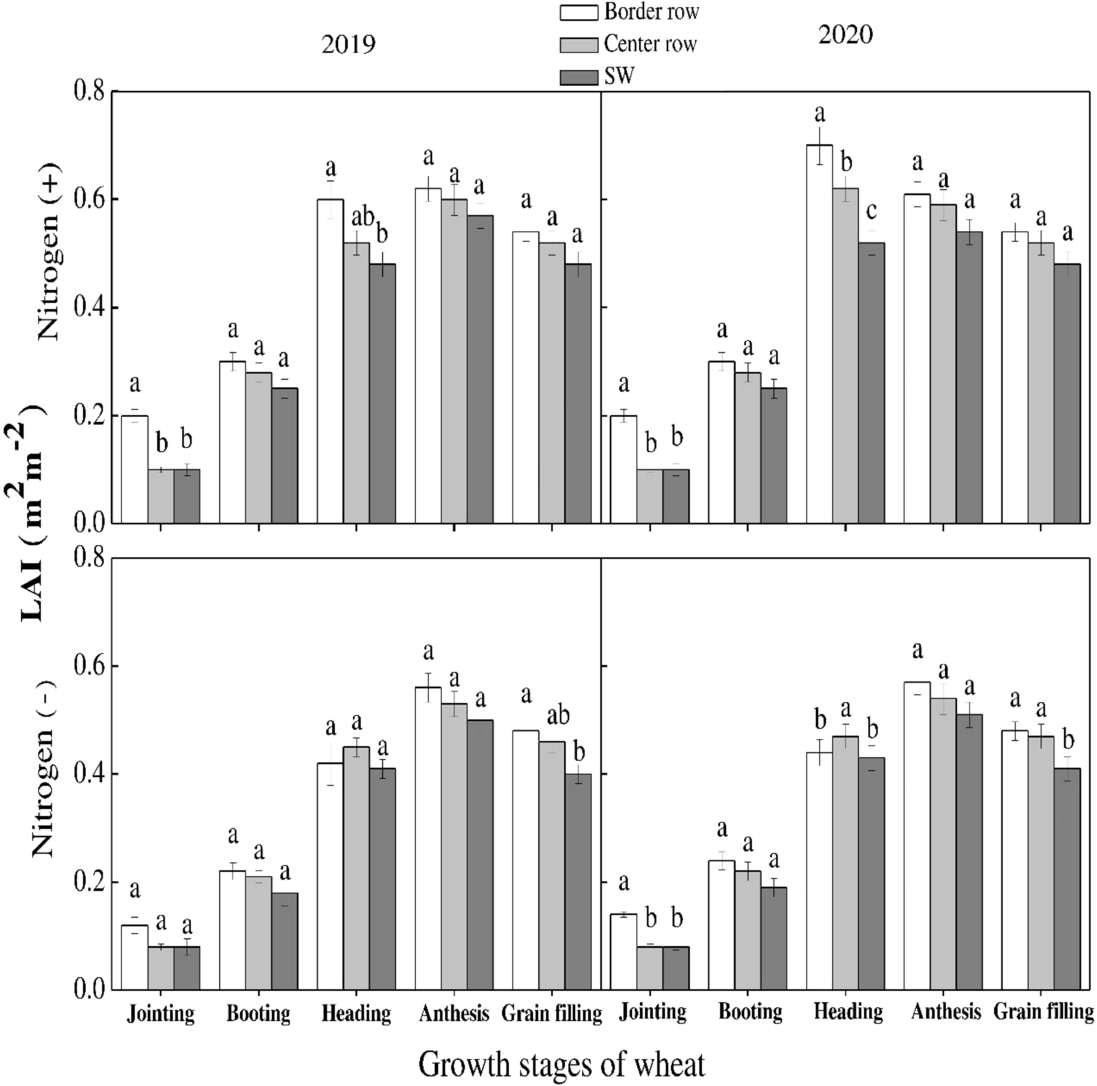
Leaf area index (LAI) of the border-, center- and sole wheat (SW) crop at various growth stages under with- and without N application. Different letters on the top of each bar indicate significant differences among the various crop growth stages, as calculated by Tukey’s HSD test at P≤ 0.05.

Leaf area index varied significantly among wheat and maize rows at various growth stages with and without N application. In maize, the leaf area index increased linearly with the passage of time till the VT stage. It started declining thereafter (**Figure 5**). At V3 and V6 stages, maximum values were recorded for sole maize and center rows in 2019 and 2020, respectively. However, an opposite trend was recorded at lateral stages at which maximum values were recorded for central rows followed by border rows while minimum was recorded for sole maize rows. For wheat crop, there was non-significant difference for leaf area index among the crop rows under both N treatments and during both study years, except for jointing stage during both years and heading stage in 2020. At jointing stage, border rows promoted leaf area index during both years (**Figure 6**). At heading stage, border rows and center rows recorded the highest values in 2019 and 2020, respectively. Results revealed that N application significantly promoted leaf area index in maize and wheat in comparison to no N application.

### 3.3 Photosynthesis, stomatal conductance, transpiration, and water use efficiency

**Figure 7:**
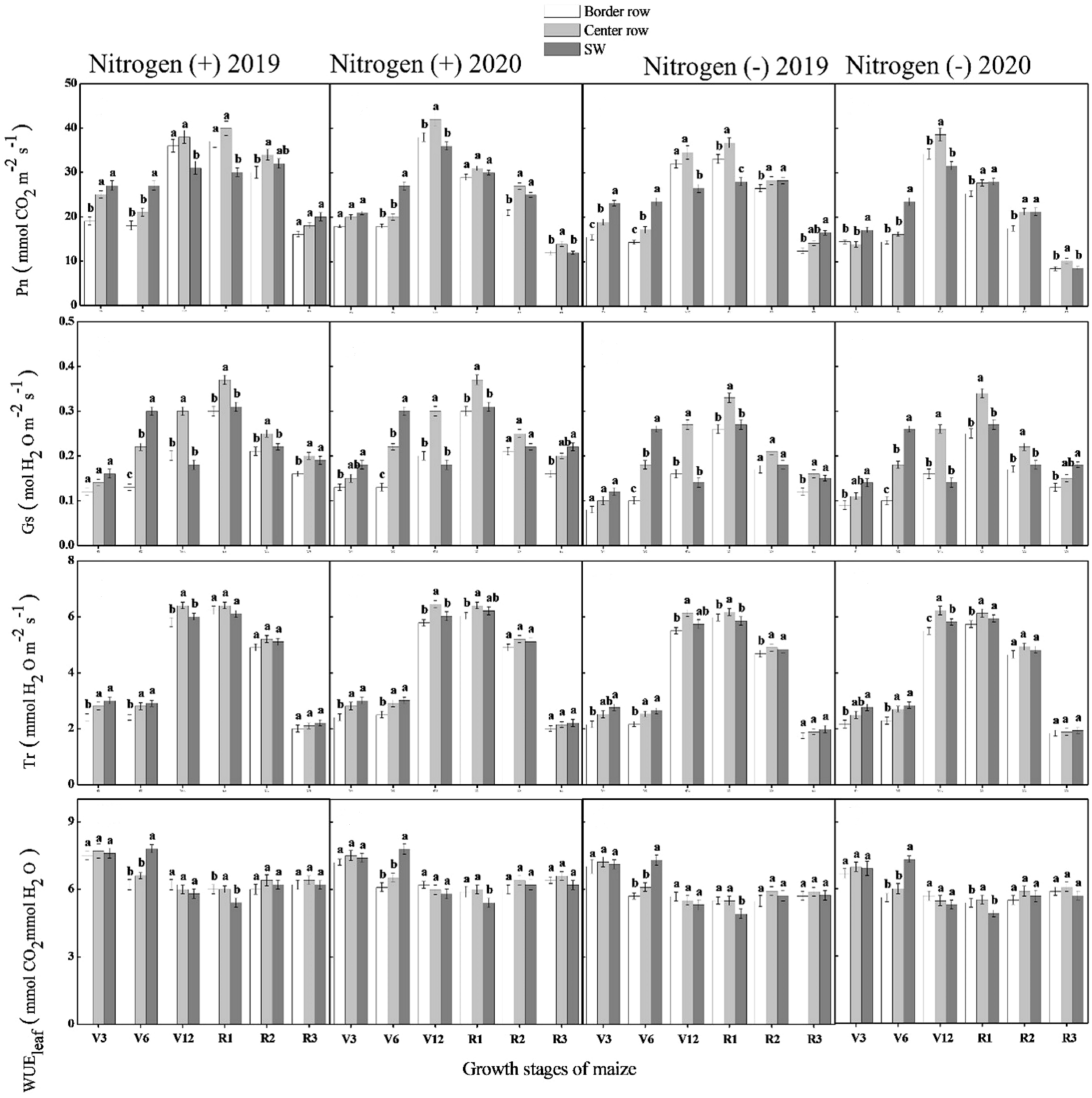
Photosynthesis rate (Pn), stomatal conductance (Gs), transpiration rate (Tr) and leaf water use efficiency (WUEleaf) in the border-, center- and sole maize (SM) crop at various growth stages under with- and without N application. Different letters on the top of each bar indicate significant differences among the various crop growth stages, as calculated by Tukey’s HSD test at P ≤ 0.05.

**Figure 8:**
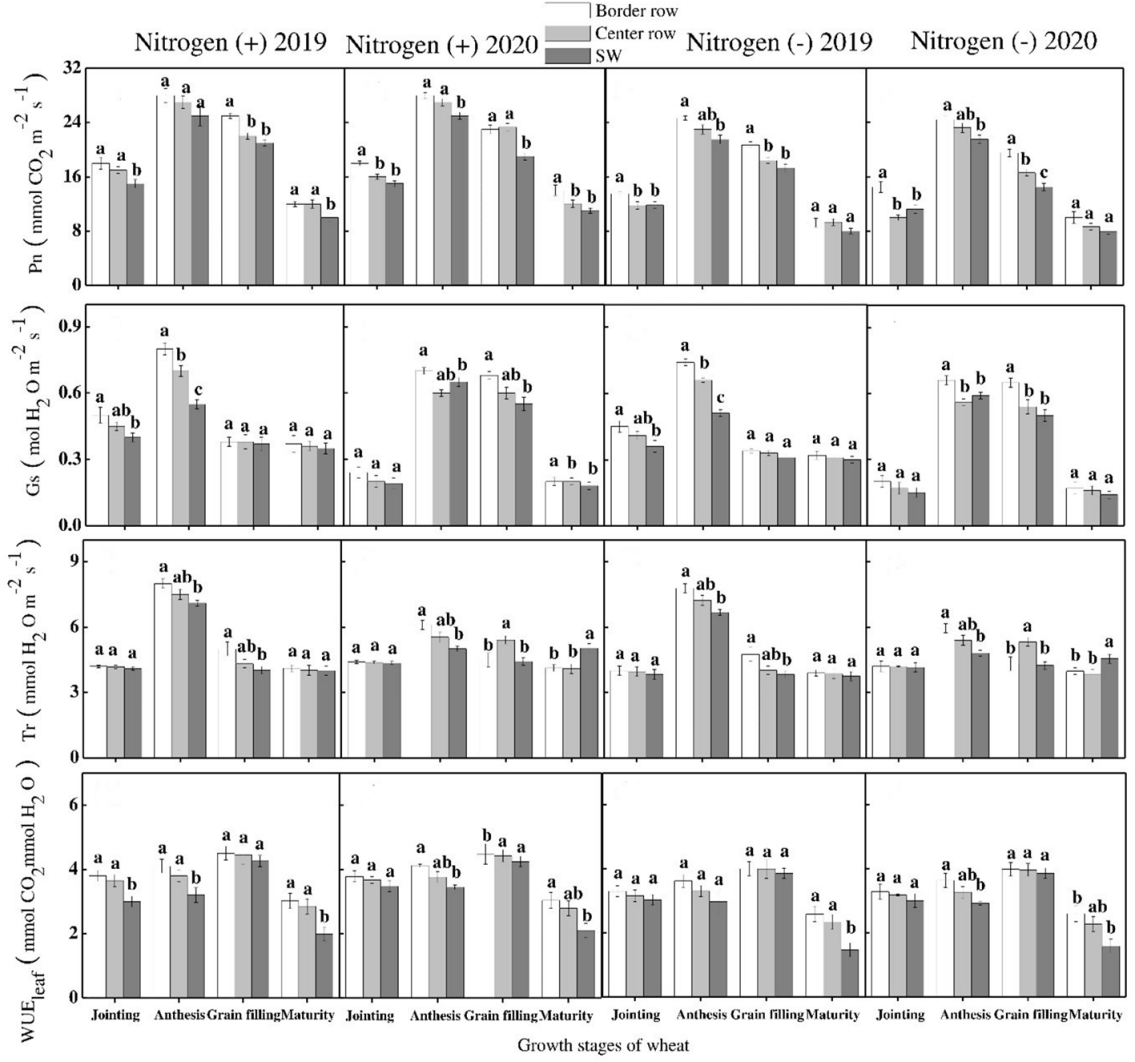
Photosynthesis rate (Pn), stomatal conductance (Gs), transpiration rate (Tr) and leaf water use efficiency (WUEleaf) in the border-, center- and sole wheat (SW) crop at various growth stages under with- and without N application. Different letters on the top of each bar indicate significant differences among the various crop growth stages, as calculated by Tukey’s HSD test at P ≤ 0.05.

There was a significant difference among crop rows, growth stages and N application in maize and wheat for net photosynthesis (Pn), transpiration rates (Tr), stomatal conductance (Gs) and leaf water use efficiency of maize during both years. Among growth stages, maximum Pn, Gs, TR and WUE were recorded for V12, R1, V12-R1, and V3-V6 stages, respectively during both years. For crop rows, at V3 and V6 stages, maximum Pn, Gs, Tr and WUE were recorded for sole maize rows during both years. Whereas, at lateral growth stages, center maize rows depicted significantly higher values of these traits during both years except for WUE for which a non-significant difference was depicted for crop rows (**Figure 7**). For wheat crop, maximum values of these traits i.e., Pn, Gs, Tr and WUE were recorded at anthesis stage during both years. Furthermore, border rows depicted higher values of these traits at all growth stages during both years. Among N treatments, data showed that significantly higher values of the aforementioned traits were recorded for N application as compared with N absence treatment in both crops during both years of experiment (**Figure 8**).

### 3.4 SPAD values

**Figure 9:**
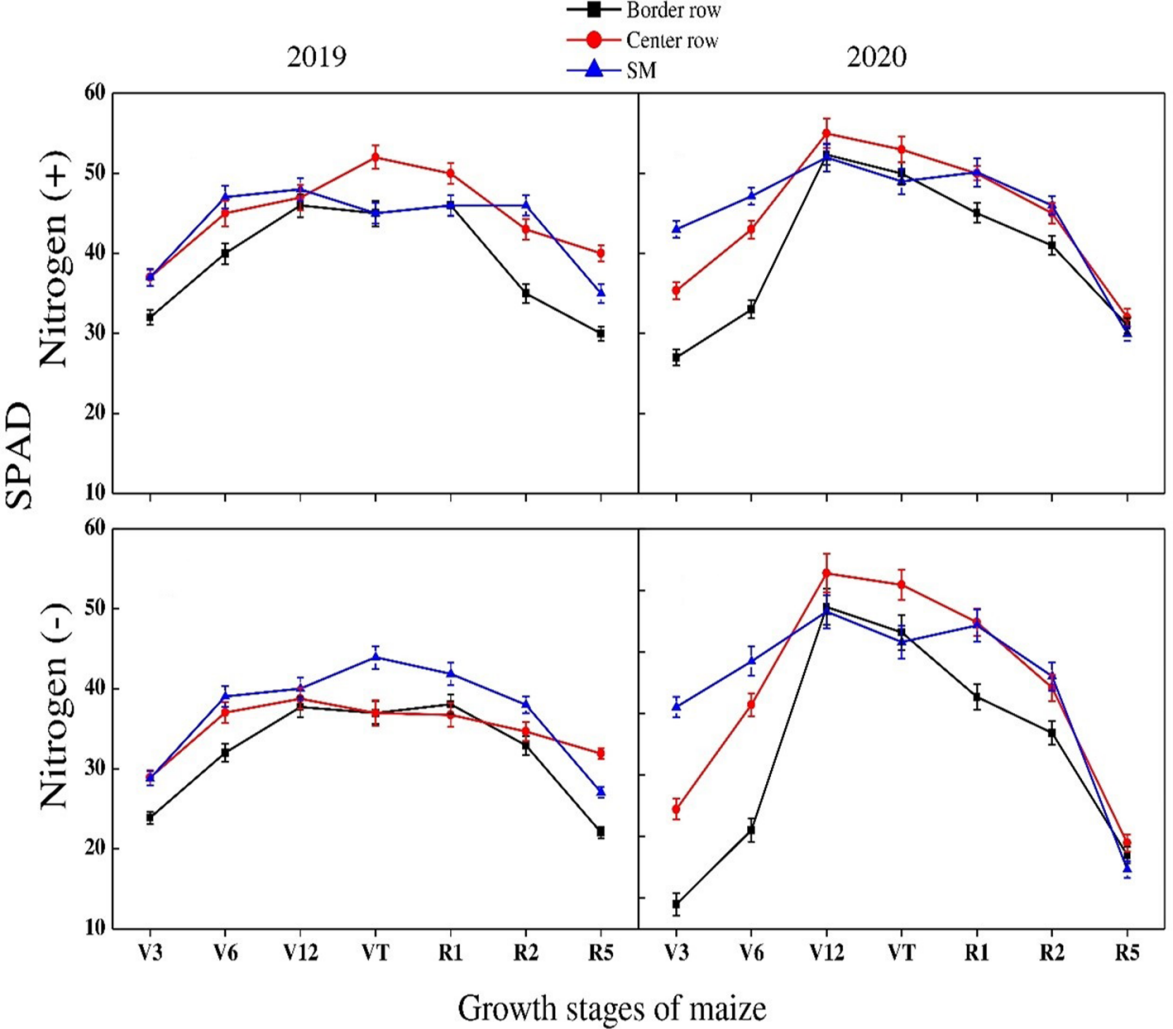
SPAD values of the border-, center- and sole maize (SM) crop at various growth stages under with- and without N application. Different letters on the top of each bar indicate significant differences among the various crop growth stages, as calculated by Tukey’s HSD test at P ≤ 0.05.

**Figure 10:**
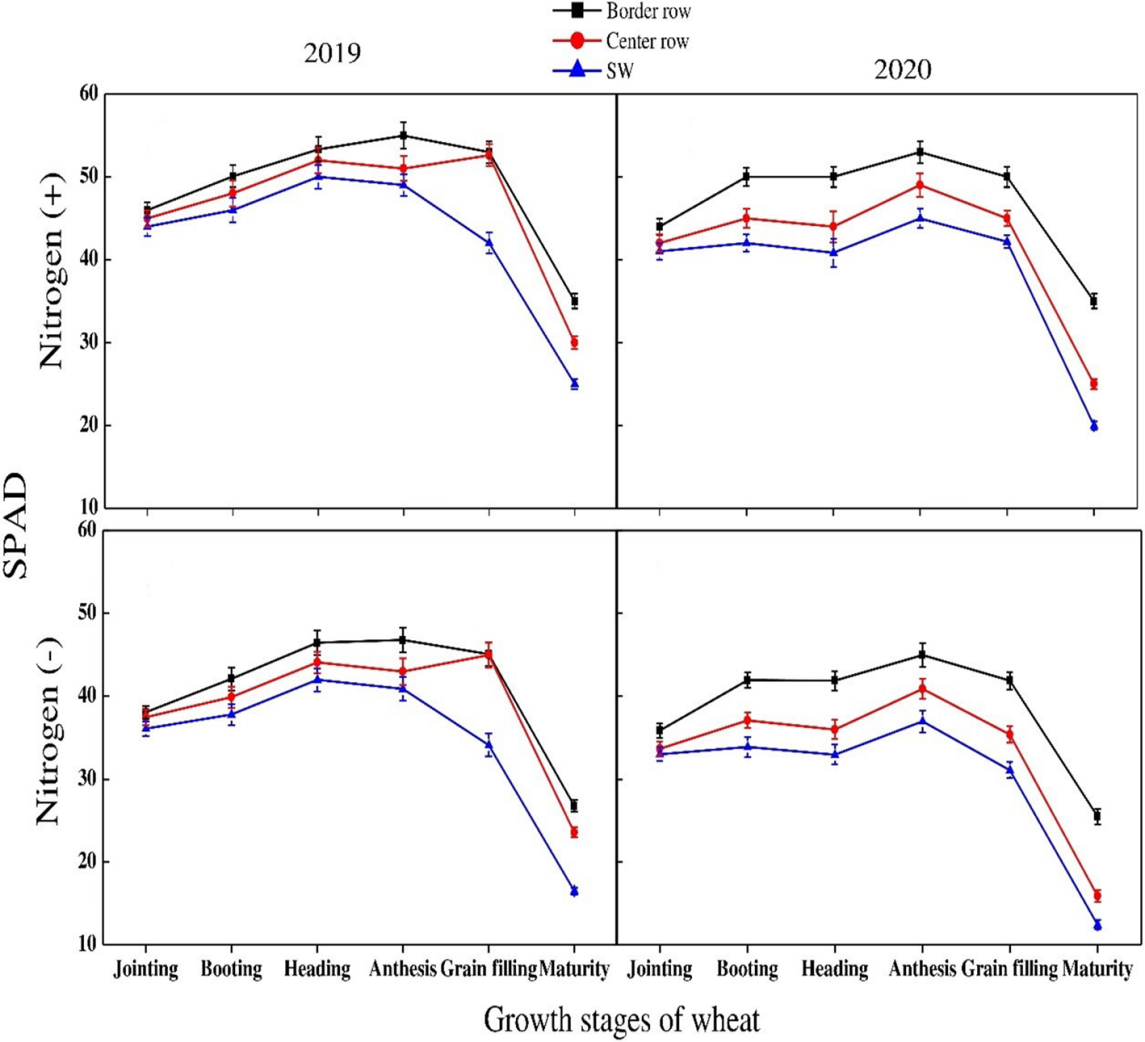
SPAD values of the border-, center- and sole wheat (SW) crop at various growth stages under with- and without N application. Different letters on the top of each bar indicate significant differences among the various crop growth stages, as calculated by Tukey’s HSD test at P ≤ 0.05.

SPAD values in maize varied significantly with varying N rates, crop growth stages and rows during both study years (**Figure 9**). For growth stages, SPAD values kept on increasing until V12 stage. These were significantly more at this stage and started decreasing thereafter. Minimum SPAD values were recorded at R5 stage. Different maize rows showed significant variation at various stages. At V3 and V6 stages, maximum values were recorded for sole maize rows. However, at V12 and VR stages, crop center rows recorded maximum values under both N treatments during both years except for N absence condition in 2019 in which maximum values were recorded for sole maize rows. At lateral growth stages, there was a non-significant difference among crop rows during both years. Similarly, there was a significant difference for SPAD values among crop rows and growth stages of wheat during both years. The SPAD values decreased with successive growth stages until the grain filling stage at which minimum values were recorded. For crop rows, data showed that maximum values were recorded for border rows at all growth stages during both years followed by center rows and sole wheat crop. Among N treatments, significantly higher values were recorded for N application when compared with no N application in both crops during both years (**Figure 10**).

### 3.5 Soil moisture storage

**Figure 11:**
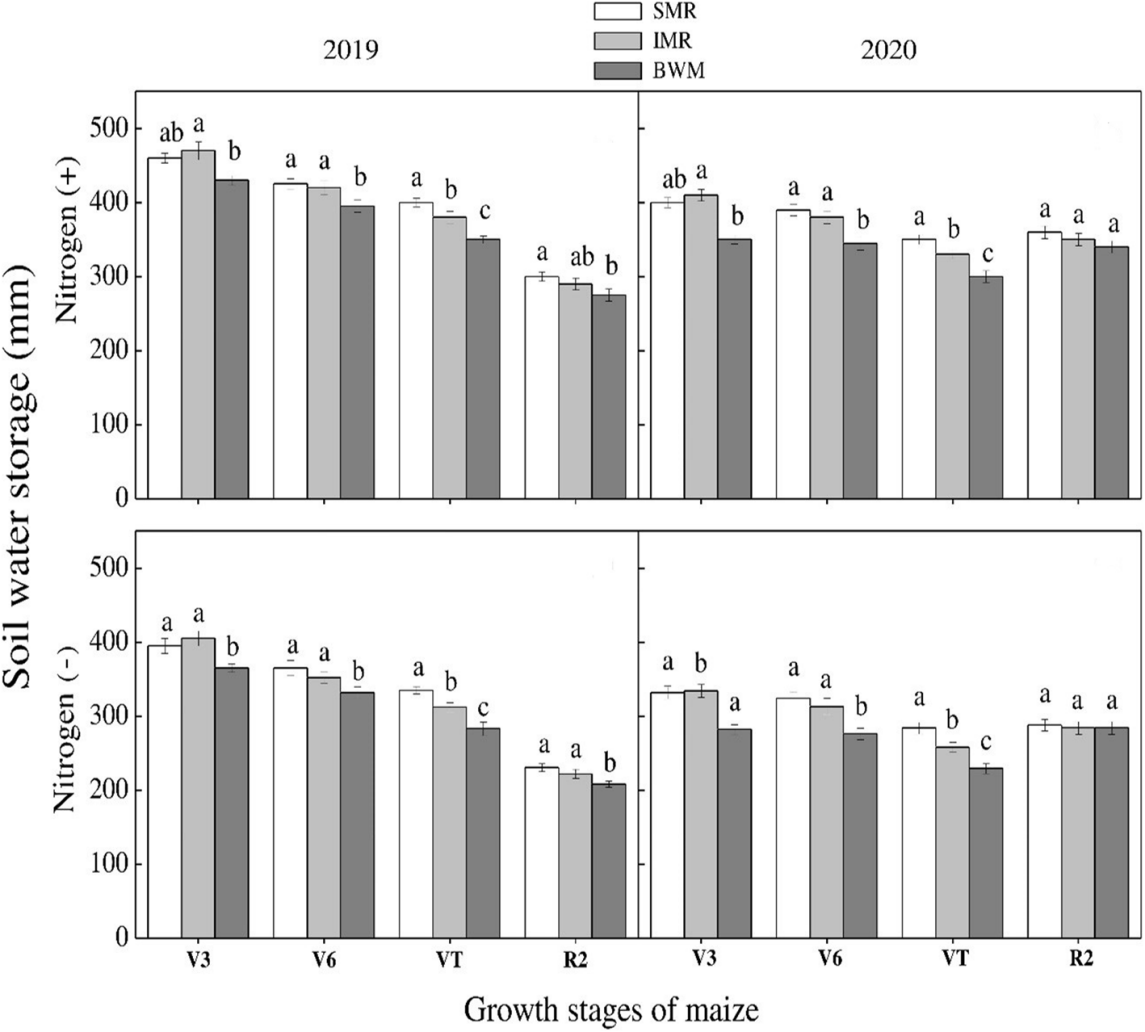
Soil water storage under sole maize row (SMR), intercropped maize row (IMR) and the rows between the intercropped strips of both crops at various growth stages under with and without N application. Different letters on the top of each bar indicate significant differences among the various crop growth stages of maize, as calculated by Tukey’s HSD test at P ≤ 0.05.

**Figure 12:**
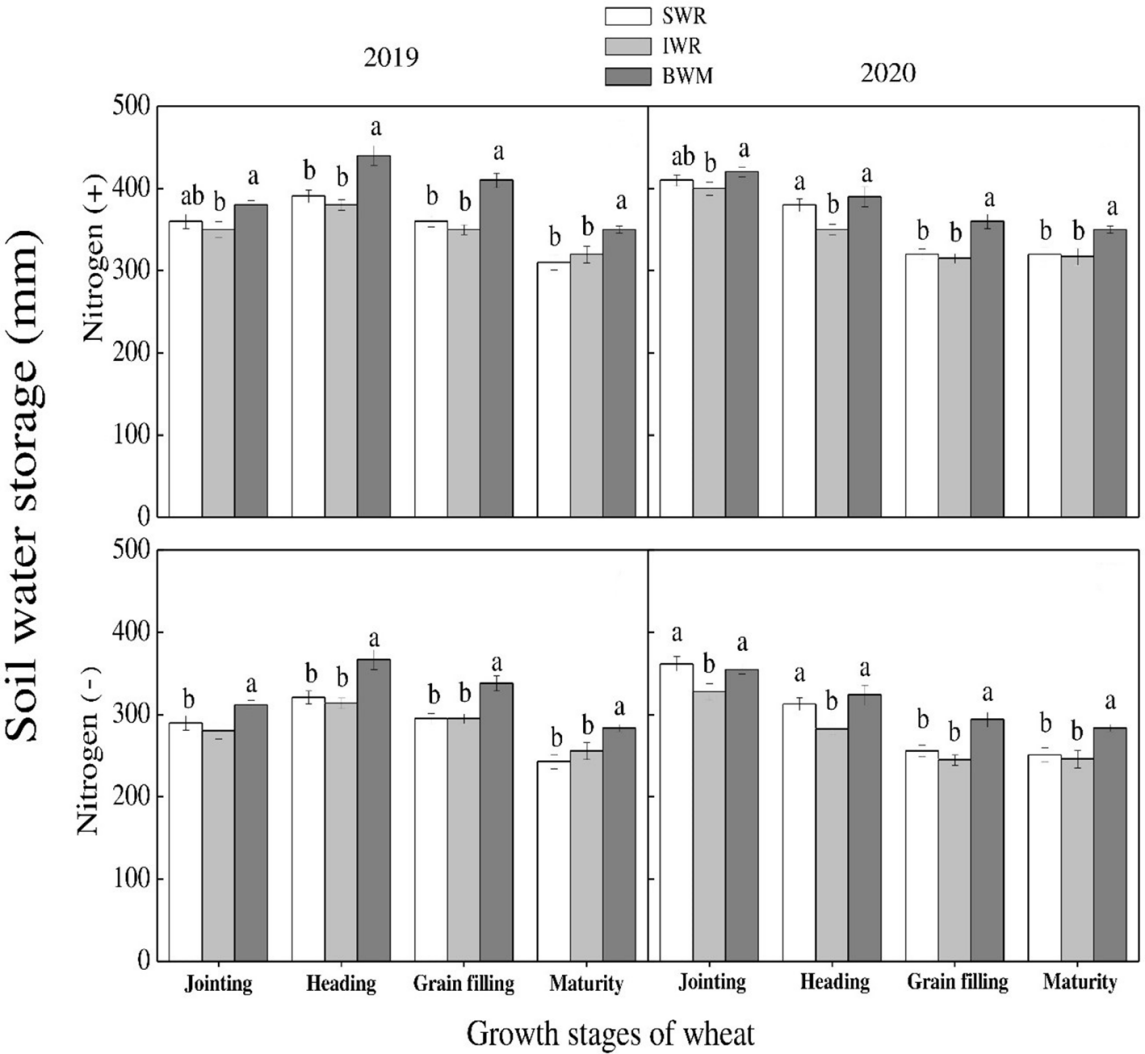
Soil water storage under sole wheat row (SWR), intercropped wheat row (IWR), and the rows between the intercropped strips of both crops (BWM) at various growth stages under with and without N application. Different letters on the top of each bar indicate significant differences among the various crop growth stages of wheat, as calculated by Tukey’s HSD test at P ≤ 0.05.

Soil moisture storage varied significantly among crop stages and N rates in maize during both years. Among growth stages, maximum values were recorded at V3 stage during both years. There was a non-significant difference among various rows at different growth stages except for VT stage. At VT stage, maximum values were recorded for border rows followed by center crop rows. Similarly, minimum values were recorded for sole maize crop (**Figure 11**). For wheat crop, there was a non-significant influence of crop rows on soil moisture storage at various stages with- and without- N application during both study years. Although the results were non-significant, the rows between the strips of both crops recorded higher values than sole and intercropped wheat rows. Among N treatments, N application significantly promoted the soil water storage during both years (**Figure 12**).

## 4 Discussion

Intercropping with balanced N application is essential for increasing crop productivity on sustainable basis, through efficient use of inputs (Chen et al., 2017). In this work, intercropping significantly improved the yield of wheat with an opposite trend to maize in which a significant decrease in yield was depicted. Furthermore, the overall grain yield of intercropped rows was greater than the monocropped rows. Previous studies have clearly demonstrated that planting geometry influences light interception and thus affects the crop productivity (Wen et al., 2019; Chapepa et al., 2020). However, further studies are needed to find out the contribution of rows of different crops to grain yield under intercropping system. In this work, under rainfed conditions and during both experimental years, the land equivalent ratio was greater than 1. This established that intercropping increased crops yield besides increasing the cropping intensity. These results are in line with previous published reports in which authors have demonstrated that intercropping system significantly promoted the crop productivity and land use efficiency under irrigated conditions (Brahimi et al., 2022; Wei et al., 2022). Our results thus clearly depict that strip intercropping promoted the overall crop yield under rainfed conditions and semi-arid climate with an average annual rainfall of about 600 mm. When compared with sole wheat crop, the higher yields of intercropped rows were mainly associated with higher numbers of ears per plant and thousand kernel weight. Higher yield related traits were mainly associated with the availability of sufficient resources for intercropped wheat before sowing of corn crop. Also, higher values might be associated with higher competitive ability of wheat crop for available resources i.e., moisture and nutrients under intercropping system. Similarly, lower maize yield under intercropping treatments was associated with lower yield-related parameters i.e., kernel number and thousand grain weight under the same treatment than that of sole maize. In line with these results, Li et al. (2001b) demonstrated that maize crop gained significantly higher growth and productivity after harvesting of wheat crop which may be associated with availability of more soil moisture and other resources. Similar results were reported in a recent study of Wang et al. (2015) in which authors reported improved yield in intercropped-maize rows than sole crop rows due to the availability of sufficient soil moisture. More recently, working with the same cropping system, a study of our group demonstrated that under intercropping system, wheat crop showed more horizontal root growth than corn crop, and resulted in less water consumption (Ma et al., 2018). Under arid regions, such type of root growth results in reduced ability of corn strips to attain full growth and reduced grain yield of intercropped strips (Ma et al., 2018). The same has been reported in current study. The results of this work, therefore, established that water scarcity is a vital growth restricting factor for maize crop, particularly under intercropping system. In line with these, working with intercropped maize, Gou et al. (2016) established that low temperature with fewer sunny days limited the growth and developmental processes of the crop. Nitrogen fertilization has been considered a dominant tool for increasing crop productivity (Hirel et al., 2001). And it is well known that higher N rates generally promote grain yield (Fan et al., 2011). Nitrogen is among the essential chlorophyll molecules which help in improving the leaf’s enzymatic activity to promote the photosynthetic process (Nasar et al., 2022). A number of previous studies have demonstrated the relationship among N application rates, photosynthetic activity, and grain yield (Sharma et al., 2019; Minhas et al., 2020). Most studies clearly mentioned that N fertilization promoted grain formation mainly by increasing the photosynthetic rates and better assimilates production (reviewed by Fernandes et al., 2022). A similar was reported in this study where N addition promoted the grain yield and related traits in both crops under sole- and combined cultivation. Furthermore, in line with previous studies, higher yield under N application was highly associated with better photosynthesis rates and chlorophyll pigment formation.

Planting geometry in terms of row arrangements influenced the yield and related parameters. For example, Li et al. (2001a) recorded significantly higher grain and biomass yields for border and next to border rows in wheat. Similar was recorded in this study where border and next to border rows recorded higher yield than others. Lower yield in other crop rows might be attributed to more shading effect and less light interception as a result of heighted plants around those rows. Higher yield in border and next-to-border rows might be due to the excessive water and nutrient uptake from the contiguous maize strip, as reported in a recent study of (Ma et al., 2018). Improved soil conditions and crop growth rates facilitate grain yield of border rows. Furthermore, our findings revealed that except the border rows and sole wheat strips, there was a non-significant difference for grain yield between the intercropped rows. This showed that increased number of rows and extended distance among crop rows may reduce the endowment of border and next-to-border row. In comparison with sole wheat strip, higher grain yield of border rows was attributed to higher values of kernel number, number of tillers and thousand grain weight. Furthermore, the rows between wheat and maize strips depicted higher values of soil water storage than sole crop strips, which may facilitate the formation of tillers. High values of number of ears in the border rows, due to availability of more moisture, were also reported by Zhu et al. (2016). In comparison with sole crop strips, higher photosynthesis rates in border rows at reproductive stage in wheat also contribute to an increase in number of grains and their weight. The results of this study are in line with Raza et al. (2022) who reported that more light availability at crop’s reproductive stages promoted the assimilates formation which in turn promoted the formation of grains. Similarly, in a recent study, Zhang et al. (2018) noted that more light interception in crop border rows increased the number and weight of grains, which ultimately promoted the overall grain yield. On the other hand, Gou et al. (2016) reported a significant decrease in wheat grain weight when grown under intercropping system. They reported this decrease was due to reduced grain filling percentage in intercropped wheat rows. It is well established from the previously published reports that photosynthesis is the foundation for biomass accumulation as well as grain formation (Panfilova et al., 2019; Zhao et al., 2021). In this work, border rows of intercropped depicted significantly higher photosynthesis rates when compared with sole crop rows, which was mainly attributed to higher nutrient uptake and light reception, as reported in previous study of Wang et al. (2017). Furthermore, current results demonstrated that border rows depicted higher values of stomatal conductance and chlorophyll content, same as reported in previous studies of Gaju et al. (2016) where authors have reported higher values of chlorophyll content associated with improved moisture uptake and light interception of border rows.

Contrary to the wheat crop, our results demonstrated lower values of yield and its components of maize border rows when compared with sole maize. However, statistically similar values for grain yield were recorded between the center and border maize rows. These findings indicated that greater number of maize rows reduced the contribution of marginal rows and ultimately reduced the maize yield reduction under intercropping system. A previous study of our group, working with the same cropping system, clearly demonstrated that during co-growth period, depleted soil moisture levels in intercropped wheat crop further created water scarce conditions for marginal corn rows (Ma et al., 2018). Similar results were reported in this work. In addition, during blended growth period, photosynthesis rates in external maize rows were lower than those of sole maize crop which may be the leading cause of decreased number of grains and their weight. In line with these, similar was reported in a previous study of Andrade et al. (1999) where authors have reported reduced number of grains per cob in marginal crop rows associated with less availability of soil moisture and reduced photosynthetic rates. Similarly, during the blended growth period, increased height of wheat crop may have caused the shading effect for the companion crop. A significant reduction in chlorophyll content of maize crop due to shading has been well reported in previous study (Naseer et al., 2022). Similarly, Pang et al. (2018) demonstrated that shading reduced CO_2_ absorption and in this way reduced the production of photosynthetic pigments. At the same time, photosynthetic rates in the external crop rows of intercropping were reduced than the sole crop strips. On the other hand, a significant recovery in photosynthetic rates has been observed after harvesting of wheat crop which might be associated with the absence of shading and improved light interception after harvesting of companion crop. Thus, from the above results, it is concluded that reduced assimilates production particularly during the blended-growth period result in lower thousand grain weight and ultimately grain yield in marginal crop rows.

Our results established that intercropping treatments and N application significantly increased the water use efficiency of both crops. A positive relation between the photosynthetic rates and leaf water use efficiency has been reported in a number of previous studies (Lawson and Vialet-Chabrand, 2019; Eyland et al., 2021). According to Ma et al. (2018), crop border rows potentially contributed to increase the leaf water use efficiency associated with high light interception. Sole wheat crop and intercropped maize recorded statistically similar values during the experimental period and border rows recorded lower water use efficiency than center crop rows. Furthermore, after wheat harvest, companion corn rows depicted higher water use efficiency. Thus, during this recovery phase, moisture conservation practices should be adapted to increase the yield of companion crop, particularly under semi-arid conditions without irrigation supplements. Freshwater shortage has emerged a worldwide problem for agricultural crops (Garrido-Cardenas et al., 2020; Yusuf et al., 2020). Intercropping has been reported as a sustainable approach for enhancing crop performance under water scares conditions (Bitew and Abera, 2019). In current work, our results demonstrated that intercropping treatments improved crop yield and overall productivity under rainfed conditions. Similarly, some other studies also reported that intercropping promoted crop/s yield and land use efficiency more than sole cropping system (Layek et al., 2018; Nasar et al., 2020). Higher yield in intercropping was attributed to improved moisture and nutrient uptake from the soil, and above-ground plant performance (Nwokoro et al., 2022; Zhao et al., 2022). In this work, there was a positive correlation for number of ears and grain yield. Therefore, the planting density can be appropriately increased to better harvest the advantage of the border rows. In conclusion, our results demonstrated that intercropping promoted the yield of wheat, however, there was a decline in intercropped maize which draw our attention to adapt suitable approaches such as cultivation of shade-tolerant cultivars, for increasing the productivity of intercropped maize. The use of organic nutrient sources including straw or biochar also helps in improving the water retaining capacity of the soil (Guo et al., 2022). These practices may contribute towards better growth of maize during recovery phase (Li et al., 2020).

## 5 Conclusions

Wheat-maize intercropping and N application promptly improved the LUE and overall grain yield in arid regions without irrigation supplements. This increase in grain yield was due to the significant improvement in wheat yield where border rows contributed maximum followed by second rows which were further attributed to the higher values for yield-related traits during both study years. Regardless of the absence of significant differences, intercropped maize, under with and without N application, recorded somewhat decrease in grain yield during both years which was mainly due to the border and second rows in which lower values were attributed to the reduced photosynthesis and chlorophyll pigmentation during the blended growth period. This study identified the greatest possibility for wheat-maize strip intercropping production improvement in rainfed areas in China and provided important information for optimizing the geometry of maize-wheat intercropping, improving regional productivity, and ensuring food security.

## Data availability statement

The original contributions presented in the study are included in the article/supplementary material. Further inquiries can be directed to the corresponding authors.

## Author contributions

SH, XC and XR planned and designed the research experiment. SH performed the experiments, collected the data and analyzed it. MN, RG, FH, SA and BA monitored the experiments and gave final shape to the manuscript. All authors contributed to the article and approved the submitted version.

## References

[B1] Alarcón-Segura V. Grass I. Breustedt G. Rohlfs M. Tscharntke T. (2022). Strip intercropping of wheat and oilseed rape enhances biodiversity and biological pest control in a conventionally managed farm scenario. J. Appl. Ecol 59:1513–23. doi: 10.1111/1365-2664.14161

[B2] Andrade F. H. Vega C. Uhart S. Cirilo A. Cantarero M. Valentinuz O. (1999). Kernel number determination in maize. Crop Sci. 39 (2), 453–459. doi: 10.2135/cropsci1999.0011183X0039000200026x

[B3] Bakhsh K. Kamran M. A. (2019). Adaptation to climate change in rain-fed farming system in punjab, Pakistan. Int. J. Commons 13 (2):833–47. doi: 10.5334/ijc.887

[B4] Bitew Y. Abera M. (2019). Conservation agriculture based annual intercropping system for sustainable crop production: A review. Indian J. Ecol. 46 (2), 235–249.

[B5] Brahimi S. Toumatia O. Drevon J. J. Zitouni A. Lazali M. (2022). Intercropping legumes and cereals increases resource use efficiency and crop productivity in low phosphorus soils under semi-arid Mediterranean conditions. Agroecol. Sustain. Food Syst. 46 (10), 1482–1501. doi: 10.1080/21683565.2022.2121951

[B6] Challinor A. J. Watson J. Lobell D. B. Howden S. M. Smith D. R. Chhetri N. (2014). A meta-analysis of crop yield under climate change and adaptation. Nat. Clim. Change 4, 287–291. doi: 10.1038/nclimate2153

[B7] Chapepa B. Mudada N. Mapuranga R. (2020). The impact of plant density and spatial arrangement on light interception on cotton crop and seed cotton yield: an overview. J. Cotton Res. 3 (1), 1–6. doi: 10.1186/s42397-020-00059-z

[B8] Chen P. Du Q. Liu X. Zhou L. I. Hussain S. Lei L. U.. (2017). Effects of reduced nitrogen inputs on crop yield and nitrogen use efficiency in a long-term maize-soybean relay strip intercropping system. PloS One 12 (9), e0184503. doi: 10.1371/journal.pone.0184503 28910355 PMC5598979

[B9] Cuartero J. Pascual J. A. Vivo J. M. Özbolat O. Sánchez-Navarro V. Egea-Cortines M.. (2022). A first-year melon/cowpea intercropping system improves soil nutrients and changes the soil microbial community. Agricult. Ecosyst. Environ. 328, 107856. doi: 10.1016/j.agee.2022.107856

[B10] David S. Vithanage M. Fraiture C. D. Faures J. M. Finlayson C. M. Gordon L.. (2011). Water availability and its use in agriculture. Treatise on water science, 707–732 doi: 10.1016/B978-0-444-53199-5.00108-1

[B11] de Sá Souza M. Júnior G. D. N. A. de Souza L. S. B. Ferraz Jardim A. M. D. R. da Silva G. I. N. de Araújo G. G. L.. (2022). Forage yield, competition and economic benefit of intercropping cactus and millet with mulch in a semi-arid environment. Afr. J. Range Forage Sci., 1–12. doi: 10.2989/10220119.2021.2016967

[B12] Dotaniya M. L. Meena V. D. Saha J. K. Dotaniya C. K. Mahmoud A. E. D. Meena B. L.. (2022). Reuse of poor-quality water for sustainable crop production in the changing scenario of climate. Environment Dev. Sustainability, 1–32. doi: 10.1007/s10668-022-02365-9 PMC9128324 35645606

[B13] Erythrina E. Susilawati S. Slameto S. Resiani N. M. D. Arianti F. D. Jumakir J.. (2022). Yield advantage and economic performance of rice–maize, rice–soybean, and maize–soybean intercropping in rainfed areas of Western Indonesia with a wet climate. Agronomy 12 (10), 2326. doi: 10.3390/agronomy12102326

[B14] Eyland D. van Wesemael J. Lawson T. Carpentier S. (2021). The impact of slow stomatal kinetics on photosynthesis and water use efficiency under fluctuating light. Plant Physiol. 186 (2), 998–1012. doi: 10.1093/plphys/kiab114 33693867 PMC8195518

[B15] Fan M. Shen J. Yuan L. Jiang R. Zhang F. (2011). Improving crop productivity and resource use efficiency to ensure food security and environmental quality in China. J. Exp. Bot. 63, 13–24. doi: 10.1093/jxb/err248 21963614

[B16] FAO IFAD UNICEF WFP WHO (2019). The state of food security and nutrition in the world 2019. safeguarding against economic slowdowns and downturns (Rome: FAO). Available at: http://www.fao.org/3/ca5162en/ca5162en.pdf.

[B17] Fernandes A. P. G. Machado J. Fernandes T. R. Vasconcelos M. W. Carvalho S. M. P. (2022). Water and nitrogen fertilization management in light of climate change: Impacts on food security and product quality. Plant Nutr. Food Secur. Era Climate Change, 147–178. doi: 10.1016/B978-0-12-822916-3.00013-5

[B18] Gaju O. DeSilva J. Carvalho P. Hawkesford M. J. Griffiths S. Greenland A.. (2016). Leaf photosynthesis and associations with grain yield, biomass and nitrogen-use efficiency in landraces, synthetic-derived lines and cultivars in wheat. Field Crops Res. 193, 1–15. doi: 10.1016/j.fcr.2016.04.018

[B19] Garrido-Cardenas J. A. Esteban-García B. Agüera A. Sánchez-Pérez J. A. Manzano-Agugliaro F. (2020). Wastewater treatment by advanced oxidation process and their worldwide research trends. Int. J. Environ. Res. Public Health 17 (1), 170. doi: 10.3390/ijerph17010170 PMC6981484 31881722

[B20] Gou F. van Ittersum M. K. Wang G. van der Putten P. E. van der Werf W. (2016). Yield and yield components of wheat and maize in wheat–maize intercropping in the Netherlands. Eur. J. Agron. 76, 17–27. doi: 10.1016/j.eja.2016.01.005

[B21] Grote U. Fasse A. Nguyen T. T. Erenstein O. (2021). Food security and the dynamics of wheat and maize value chains in Africa and Asia. Front. Sustain. Food Syst. 4, 317. doi: 10.3389/fsufs.2020.617009

[B22] Guo R. Qian R. Yang L. Khaliq A. Han F. Hussain S.. (2022). Interactive effects of maize straw-derived biochar and n fertilization on soil bulk density and porosity, maize productivity and nitrogen use efficiency in arid areas. J. Soil Sci. Plant Nutr. 22, 1–21. doi: 10.1007/s42729-022-00881-1

[B23] Hirel B. Bertin P. Quilleré I. Bourdoncle W. Attagnant C. Dellay C.. (2001). Towards a better understanding of the genetic and physiological basis for nitrogen use efficiency in maize. Plant Physiol. 125 (3), 1258–1270. doi: 10.1104/pp.125.3.1258 11244107 PMC65606

[B24] Jannoura R. Joergensen R. G. Bruns C. (2014). Organic fertilizer effects on growth, crop yield, and soil microbial biomass indices in sole and intercropped peas and oats under organic farming conditions. Eur. J. Agron. 52, 259–270. doi: 10.1016/j.eja.2013.09.001

[B25] Jo S. G. Kang Y. I. Om K. S. Cha Y. H. Ri S. Y. (2022). Growth, photosynthesis and yield of soybean in ridge-furrow intercropping system of soybean and flax. Field Crops Res. 275, 108329. doi: 10.1016/j.fcr.2021.108329

[B26] Lawson T. Vialet-Chabrand S. (2019). Speedy stomata, photosynthesis and plant water use efficiency. New Phytol. 221 (1), 93–98. doi: 10.1111/nph.15330 29987878

[B27] Layek J. Das A. Mitran T. Nath C. Meena R. S. Yadav G. S.. (2018). “Cereal+ legume intercropping: An option for improving productivity and sustaining soil health,” in Legumes for soil health and sustainable management (Singapore: Springer), 347–386.

[B28] Li Y. Ma L. Wu P. Zhao X. Chen X. Gao X. (2020). Yield, yield attributes and photosynthetic physiological characteristics of dryland wheat (Triticum aestivum l.)/maize (Zea mays l.) strip intercropping. Field Crops Res. 248, 107656. doi: 10.1016/j.fcr.2019.107656

[B29] Li L. Sun J. Zhang F. Li X. Rengel Z. Yang S. (2001b). Wheat/maize or wheat/soybean strip intercropping: II. recovery or compensation of maize and soybean after wheat harvesting. Field Crops Res. 71 (3), 173–181. doi: 10.1016/S0378-4290(01)00157-5

[B30] Li L. Sun J. Zhang F. Li X. Yang S. Rengel Z. (2001a). Wheat/maize or wheat/soybean strip intercropping: I. yield advantage and interspecific interactions on nutrients. Field Crops Res. 71 (2), 123–137. doi: 10.1016/S0378-4290(01)00156-3

[B31] Li S. van der Werf W. Zhu J. Guo Y. Li B. Ma Y.. (2021). Estimating the contribution of plant traits to light partitioning in simultaneous maize/soybean intercropping. J. Exp. Bot. 72 (10), 3630–3646. doi: 10.1093/jxb/erab077 33608704

[B32] Liang Y. F. Khan S. Ren A. X. Lin W. Anwar S. Sun M.. (2019). Subsoiling and sowing time influence soil water content, nitrogen translocation and yield of dryland winter wheat. Agronomy 9 (1), 37. doi: 10.3390/agronomy9010037

[B33] Ma L. Li Y. Wu P. Zhao X. Chen X. Gao X. (2019). Effects of varied water regimes on root development and its relations with soil water under wheat/maize intercropping system. Plant Soil 439 (1), 113–130. doi: 10.1007/s11104-018-3800-9

[B34] Ma L. Li Y. Wu P. Zhao X. Chen X. Gao X. (2020). Coupling evapotranspiration partitioning with water migration to identify the water consumption characteristics of wheat and maize in an intercropping system. Agricultural and Forest Meteorology 290, 108034

[B35] Maitra S. Hossain A. Brestic M. Skalicky M. Ondrisik P. Gitari H.. (2021). Intercropping–a low input agricultural strategy for food and environmental security. Agronomy 11 (2), 343. doi: 10.3390/agronomy11020343

[B36] Minhas W. A. Hussain M. Mehboob N. Nawaz A. UL-Allah S. Rizwan M. S.. (2020). Synergetic use of biochar and synthetic nitrogen and phosphorus fertilizers to improves maize productivity and nutrient retention in loamy soil. J. Plant Nutr. 43 (9), 1356–1368. doi: 10.1080/01904167.2020.1729804

[B37] Nasar J. Shao Z. Arshad A. Jones F. G. Liu S. Li C.. (2020). The effect of maize–alfalfa intercropping on the physiological characteristics, nitrogen uptake and yield of maize. Plant Biol. 22 (6), 1140–1149. doi: 10.1111/plb.13157 32609937

[B38] Nasar J. Wang G. Y. Ahmad S. Muhammad I. Zeeshan M. Gitari H.. (2022). Nitrogen fertilization coupled with iron foliar application improves the photosynthetic characteristics, photosynthetic nitrogen use efficiency, and the related enzymes of maize crops under different planting patterns. Front. Plant Sci. 13, 988055. doi: 10.3389/fpls.2022.988055 36119633 PMC9478416

[B39] Naseer M. A. Hussain S. Nengyan Z. Ejaz I. Ahmad S. Farooq M.. (2022). Shading under drought stress during grain filling attenuates photosynthesis, grain yield and quality of winter wheat in the loess plateau of China. J. Agron. Crop Sci. 208 (2), 255–263. doi: 10.1111/jac.12563

[B40] Nwokoro C. C. Kreye C. Necpalova M. Adeyemi O. Barthel M. Pypers P.. (2022). Cassava-maize intercropping systems in southern Nigeria: Radiation use efficiency, soil moisture dynamics, and yields of component crops. Field Crops Res. 283, 108550. doi: 10.1016/j.fcr.2022.108550 35782166 PMC9133798

[B41] Obi E. A. Agele S. O. Aiyelari O. P. Adejoro S. A. Agbona A. I. (2022). Nutrient uptake and use efficiencies of strip intercropped cassava, maize and pepper as affected by fertilizer type and age of oil palm fields in an oil palm-based intercropping system. J. Soil Sci. Environ. Manage. 13 (2), 23–35. doi: 10.5897/JSSEM2020.0818

[B42] Panfilova A. Korkhova M. Gamayunova V. Drobitko A. Nikonchuk N. Markova N. (2019). Formation of photosynthetic and grain yield of soft winter wheat (Triticum aestivum l.) depending on varietal characteristics and optimization of nutrition. Research journal of pharmaceutical, biological and chemical sciences 10 (2), 78–85. Available at: http://dspace.mnau.edu.ua/jspui/handle/123456789/5698.

[B43] Pang R. Sun Y. Xu X. Song M. Ouyang H. (2018). Effects of clipping and shading on 15NO3– and 15NH4+ recovery by plants in grazed and ungrazed temperate grasslands. Plant Soil 433 (1), 339–352. doi: 10.1007/s11104-018-3844-x

[B44] Raza M. A. Gul H. Hasnain A. Khalid M. H. B. Hussain S. Abbas G.. (2022). Leaf area regulates the growth rates and seed yield of soybean (Glycine max l. merr.) in intercropping system. Int. J. Plant Production 16, 635–52. doi: 10.1007/s42106-022-00201-8

[B45] Roohi M. Arif M. S. Guillaume T. Yasmeen T. Riaz M. Shakoor A.. (2022). Role of fertilization regime on soil carbon sequestration and crop yield in a maize-cowpea intercropping system on low fertility soils. Geoderma 428, 116152. doi: 10.1016/j.geoderma.2022.116152

[B46] Sharma R. Adhikari P. Shrestha J. Acharya B. P. (2019). Response of maize (Zea mays l.) hybrids to different levels of nitrogen. Arch. Agric. Environ. Sci. 4 (3), 295–299. doi: 10.26832/24566632.2019.040306

[B47] UNESCO (2009). The united nations world water development report 3: Water in a changing world (Earth scan, Paris: UNESCO, and London).

[B48] Vågsholm I. Arzoomand N. S. Boqvist S. (2020). Food security, safety, and sustainability–getting the trade-offs right. Front. Sustain. Food Syst. 4, 16. doi: 10.3389/fsufs.2020.00016

[B49] Varis O. Keskinen M. Kummu M. (2017). Four dimensions of water security with a case of the indirect role of water in global food security. Water Secur. 1, 36–45. doi: 10.1016/j.wasec.2017.06.002

[B50] Waha K. Dietrich J. P. Portmann F. T. Siebert S. Thornton P. K. Bondeau A.. (2020). Multiple cropping systems of the world and the potential for increasing cropping intensity. Global Environ. Change 64, 102131. doi: 10.1016/j.gloenvcha.2020.102131 PMC7737095 33343102

[B51] Wang J. Hussain S. Sun X. Zhang P. Javed T. Dessoky E. S.. (2022). Effects of nitrogen application rate under straw incorporation on photosynthesis, productivity and nitrogen use efficiency in winter wheat. Frontiers in Plant Science 13. 10.3389/fpls.2022.862088 PMC8966908 35371129

[B52] Wang R. Sun Z. Bai W. Wang E. Wang Q. Zhang D.. (2021). Canopy heterogeneity with border-row proportion affects light interception and use efficiency in maize/peanut strip intercropping. Field Crops Res. 271, 108239. doi: 10.1016/j.fcr.2021.108239

[B53] Wang Z. Zhao X. Wu P. Chen X. (2015). Effects of water limitation on yield advantage and water use in wheat (Triticum aestivum l.)/maize (Zea mays l.) strip intercropping. Eur. J. Agron. 71, 149–159. doi: 10.1016/j.eja.2015.09.007

[B54] Wani S. P. Sreedevi T. K. Rockström J. Ramakrishna Y. S. (2009). Rainfed agriculture–past trends and future prospects. Rainfed agriculture: Unlocking potential 7, 1–33. doi: 10.1079/9781845933890.0001

[B55] Wei W. Liu T. Shen L. Wang X. Zhang S. Zhang W. (2022). Effect of maize (Zeal mays) and soybean (Glycine max) intercropping on yield and root development in xinjiang, China. Agriculture 12 (7), 996. doi: 10.3390/agriculture12070996

[B56] Wen W. Guo X. Li B. Wang C. Wang Y. Yu Z.. (2019). Estimating canopy gap fraction and diffuse light interception in 3D maize canopy using hierarchical hemispheres. Agric. For. Meteorol. 276, 107594. doi: 10.1016/j.agrformet.2019.05.025

[B57] Wolińska A. Kruczyńska A. Podlewski J. Słomczewski A. Grządziel J. Gałązka A.. (2022). Does the use of an intercropping mixture really improve the biology of monocultural soils? a search for bacterial indicators of sensitivity and resistance to long-term maize monoculture. Agronomy 12 (3), 613. doi: 10.3390/agronomy12030613

[B58] Yang Y. Li Y. Mei X. Yang M. Huang H. Du F.. (2022). Antimicrobial terpenes suppressed the infection process of phytophthora in fennel-pepper intercropping system. Front. Plant Sci. 13, 890534. doi: 10.3389/fpls.2022.890534 35755704 PMC9218821

[B59] Yu Y. Stomph T. J. Makowski D. van der Werf W. (2015). Temporal niche differentiation increases the land equivalent ratio of annual intercrops: a meta-analysis. Field Crops Res. 184, 133–144. doi: 10.1016/j.fcr.2015.09.010

[B60] Yusuf A. Sodiq A. Giwa A. Eke J. Pikuda O. De Luca G.. (2020). A review of emerging trends in membrane science and technology for sustainable water treatment. J. cleaner production 266, 121867. doi: 10.1016/j.jclepro.2020.121867

[B61] Zhang C. J. Chu H. J. Chen G. X. Shi D. W. Zuo M. Wang J.. (2007). Photosynthetic and biochemical activities in flag leaves of a newly developed super high-yield hybrid rice (Oryza sativa) and its parents during the reproductive stage. J. Plant Res. 120, 209–217. doi: 10.1007/s10265-006-0038-z 17077941

[B62] Zhang D. Du G. Sun Z. Bai W. Wang Q. Feng L.. (2018). Agroforestry enables high efficiency of light capture, photosynthesis and dry matter production in a semi-arid climate. Eur. J. Agron. 94, 1–11. doi: 10.1016/j.eja.2018.01.001

[B63] Zhang R. Meng L. Li Y. Wang X. Ogundeji A. O. Li X.. (2021). Yield and nutrient uptake dissected through complementarity and selection effects in the maize/soybean intercropping. Food Energy Secur. 10 (2), 379–393. doi: 10.1002/fes3.282

[B64] Zhao X. Dong Q. Han Y. Zhang K. Shi X. Yang X.. (2022). Maize/peanut intercropping improves nutrient uptake of side-row maize and system microbial community diversity. BMC Microbiol. 22 (1), 1–16. doi: 10.1186/s12866-021-02425-6 34996375 PMC8740425

[B65] Zhao B. Ma B. L. Hu Y. Liu J. (2021). Source–sink adjustment: A mechanistic understanding of the timing and severity of drought stress on photosynthesis and grain yields of two contrasting oat (Avena sativa l.) genotypes. J. Plant Growth Regul. 40 (1), 263–276. doi: 10.1007/s00344-020-10093-5

[B66] Zhu J. van der Werf W. Vos J. Anten N. P. R. van der Putten P. E. L. Evers J. B. (2016). High productivity of wheat intercropped with maize is associated with plant architectural responses. Ann. Appl. Biol. 168 (3), 357–372. doi: 10.1111/aab.12268

[B67] Zhu S. G. Zhu H. Cheng Z. G. Zhou R. Yang Y. M. Wang J.. (2022). Soil water and phosphorus availability determines plant-plant facilitation in maize-grass pea intercropping system. Plant Soil 482, 451–467. doi: 10.1007/s11104-021-05280-6

